# Modulation of Protein Fouling and Interfacial Properties at Carbon Surfaces via Immobilization of Glycans Using Aryldiazonium Chemistry

**DOI:** 10.1038/srep24840

**Published:** 2016-04-25

**Authors:** Federico Zen, M. Daniela Angione, James A. Behan, Ronan J. Cullen, Thomas Duff, Joana M. Vasconcelos, Eoin M. Scanlan, Paula E. Colavita

**Affiliations:** 1 School of Chemistry and Centre for Research on Adaptive Nanostructures and Nanodevices (CRANN), Trinity College Dublin, College Green, Dublin 2, Ireland

## Abstract

Carbon materials and nanomaterials are of great interest for biological applications such as implantable devices and nanoparticle vectors, however, to realize their potential it is critical to control formation and composition of the protein corona in biological media. In this work, protein adsorption studies were carried out at carbon surfaces functionalized with aryldiazonium layers bearing mono- and di-saccharide glycosides. Surface IR reflectance absorption spectroscopy and quartz crystal microbalance were used to study adsorption of albumin, lysozyme and fibrinogen. Protein adsorption was found to decrease by 30–90% with respect to bare carbon surfaces; notably, enhanced rejection was observed in the case of the tested di-saccharide vs. simple mono-saccharides for near-physiological protein concentration values. ζ-potential measurements revealed that aryldiazonium chemistry results in the immobilization of phenylglycosides without a change in surface charge density, which is known to be important for protein adsorption. Multisolvent contact angle measurements were used to calculate surface free energy and acid-base polar components of bare and modified surfaces based on the van Oss-Chaudhury-Good model: results indicate that protein resistance in these phenylglycoside layers correlates positively with wetting behavior and Lewis basicity.

Much effort towards the design and fabrication of biomaterials and medical devices is dedicated to the attainment of desirable surface chemistry and surface physical properties, as these can often determine the biological response to materials *in vivo*^1^. There is therefore a strong interest in investigating surface modification strategies that enable a degree of control over interfacial biointeractions. Protein-surface interactions are thought to be of particular importance due to the abundance of these molecules in tissues and biological fluids and due to the central role of peptides and proteins in cell adhesion and signalling. Depending on the specific biomaterial and its application (e.g. biosensor, implant) it might be desirable to either promote protein adsorption or repel protein build-up in order to modulate performance^2^,^3^,^4^,^5^. Therefore, much effort has been devoted to developing surface modification strategies to modulate protein-surface interactions.

Various forms of carbon find multiple applications as biomaterials; coatings such as pyrocarbon and amorphous carbons (e.g. a-C, a-C:Si, a-C:H, ta-C)^6^,^7^, are promising for biomedical applications because of their frictional and mechanical properties, their corrosion resistance and chemical inertness, and their bio- and hemocompatibility. Carbon nanomaterials, such as nanotubes and nanodiamonds, have also received much attention as delivery agents for *in vivo* imaging and sensing^8^,^9^. Finally, materials such as diamond electrodes, carbon coatings and carbon nanofibers are routinely used for *in vivo* and *in vitro* bioanalytical chemistry^10^,^11^. For all of these applications it is critical to achieve control over interfacial interactions of the carbon solid surface with proteins in solution, to avoid unspecific adsorption that might result in undesirable cell-surface events, or in blocking of sensing/binding sites^12^,^13^,^14^,^15^.

Several surface modification methods have been investigated in order to control and minimize protein fouling at surfaces: cationic polymers, enzymes or peptides are effective but costly and often present problems of leaching and durability^16^. Poly and oligo (ethylene glycol) (PEG, OEG) coatings have been shown to successfully minimize protein adsorption^12^,^17^; however, PEG/OEGs can easily oxidize, losing their antifouling properties^16^. This problem has prompted a search for alternative antifouling coatings with enhanced chemical stability. In an effort to mimic biological antifouling strategies, work has focused on the use of immobilized carbohydrates, given the presence of these molecules in the antiadhesive glycocalyx that surrounds certain cells^18^,^19^. Research shows, in fact, that oligo- and polysaccharide coatings can control fouling and protein adsorption, while being extremely stable to oxidation^20^,^21^,^22^,^23^,^24^,^25^,^26^.

The use of aryldiazonium salt chemistry for the immobilization of simple carbohydrates on carbon surfaces was recently reported by our group^27^. Aryldiazonium chemistry offers a versatile route for surface immobilization with key advantages for carbon applications: (a) functionalization can be carried out from solution, (b) it occurs under mild conditions without the use of multistep reactions, and (c) it leads to the formation of robust functional layers via formation of strong C—C covalent bonds between R-Ph groups and carbon substrates^28^. This is a desirable property that imparts chemical and thermal stability to carbohydrate adlayers under a variety of conditions thus preventing interfacial exchange between the layer and biomolecules in solution. The ability to solution process surfaces also makes it intrinsically scalable and thus relevant for widespread applications. We have recently shown that immobilized phenylglycosides bearing mono-saccharide groups obtained via aryldiazonium chemistry can reduce the unspecific adsorption of Bovine Serum Albumin (BSA) at carbon surfaces^27^. However, it remains unclear whether antifouling properties can be observed with other proteins and whether specific carbohydrate structural properties are responsible for the antifouling behavior. Interestingly, we have also identified that phenyl-lactosides are more effective than mono-saccharide glycosides at preventing adsorption on polymer surfaces^20^.

Herein, we report a study of protein adsorption at phenylglycoside-modified and bare amorphous carbon surfaces using five different glycosides, four bearing mono-saccharide moieties and one being a phenyl-lactoside. We use three proteins with different levels of structural complexity and isoelectric points to understand the generality of protein adsorption trends. Importantly, we investigate the relationship between protein adsorption at phenylglycoside layers and surface free energy, charge and glycoside structure with the aim of improving our current understanding of key properties that result in antifouling activity of aryldiazonium carbohydrate layers.

## Results

### Protein adsorption studies

Amorphous carbon (a-C) films used in our experiments were deposited via magnetron sputtering. These films had previously been characterized via a combination of spectroscopic methods^29^. Briefly, they consist of approximately 80% trigonally bonded carbon (sp^2^ centers), as estimated via X-ray photoelectron spectroscopy (XPS) and Raman spectroscopy. The films also contain oxidized groups resulting in a 9% O/C atomic ratio as determined via XPS.

**Figure 1:**
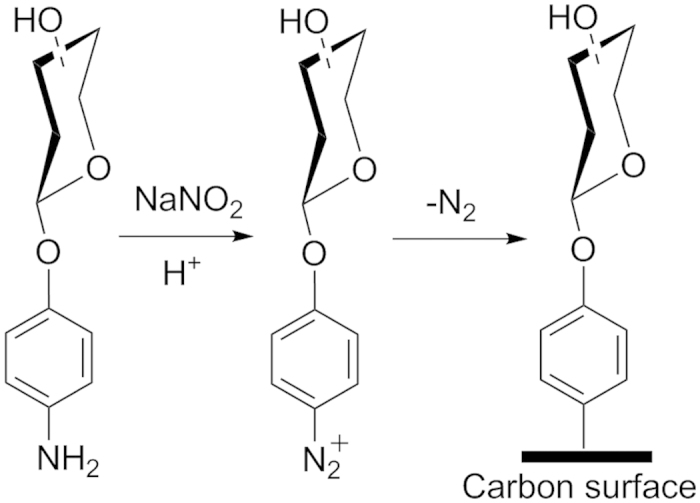
Surface modification reaction for carbon surfaces via *in situ* generation of aryldiazonium salts.

**Figure 2:**
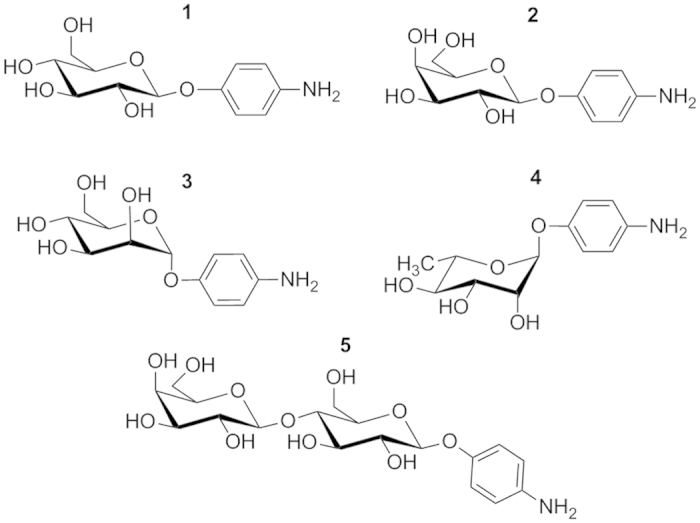
4-aminophenyl glycosides synthesized as precursors for the preparation of carbohydrate layers via aryldiazonium chemistry: 4-aminophenol-β-D-glucopyranose (**1**), 4-aminophenol-β-D-galactopyranose (**2**), 4-aminophenol-α-D-mannopyranose (**3**), 4-aminophenol-α-L-rhamnopyranose (**4**) and 4-aminophenol-β-D-lactopyranose (**5**).

**Figure 3:**
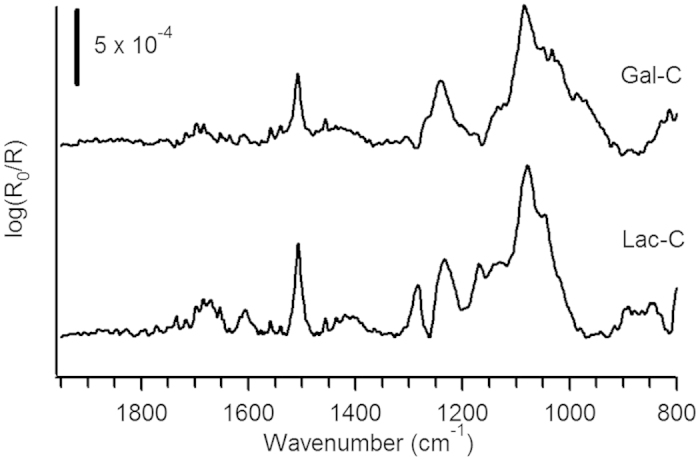
IRRAS spectra of a-C surfaces after modification with Gal (Gal-C) and Lac monosaccharides (Lac-C).

Modification of a-C with aryldiazonium salts was carried out as in our previous work (Fig. 1), via diazoniation of 4-aminophenyl glycoside precursors *in situ*. Precursor glycosides bearing glucose (Glc), galactose (Gal), mannose (Man), rhamnose (Rha) and lactose (Lac) groups (compounds **1–5**, Fig. 2), yielded surfaces from here onwards referred to as Glc-C, Gal-C, Man-C, Rha-C and Lac-C, respectively. Figure 3 shows examples of IR reflectance absorption spectroscopy (IRRAS) of Gal-C, a monosaccharide-modified surface, and of Lac-C, a disaccharide-modified surface, obtained from precursors **2** and **5**, respectively. Both IRRAS spectra show the characteristic infrared absorbances of glycosides in the region 1290–950 cm^−1^ due to C–O stretching modes arising from the carbohydrate ring^27^,^30^. Peaks in the region 1550–1500 cm^−1^ arise from C–C skeletal vibrations of phenyl rings^30^; in particular, it was possible to observe in all spectra the presence of a peak at 1508 cm^−1^ which can be attributed to the strong 19a stretching mode of phenyl rings^27^. Similar IRRAS spectra were obtained for Glc-C, Man-C and Rha-C surfaces.

**Figure 4:**
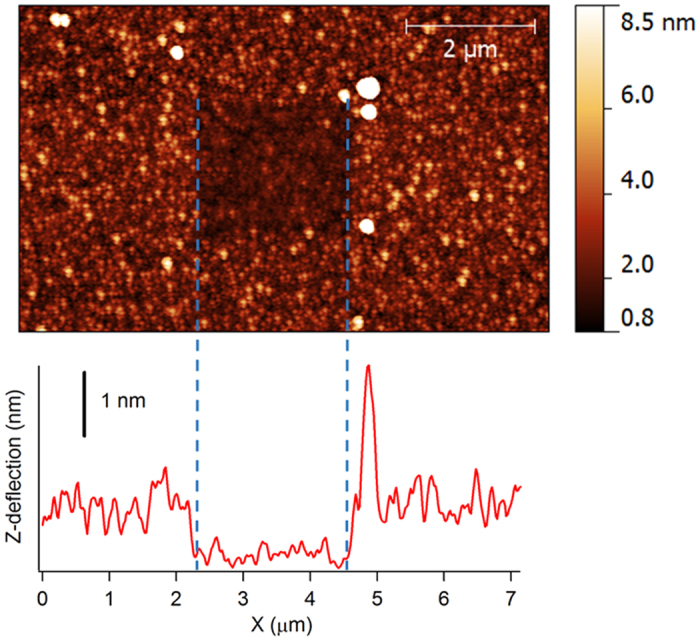
AFM topographic image of a Lac-C surface (top) after removal of a portion of the film with the AFM tip. The height profile (bottom) shows a step edge with a height equivalent to the thickness of the phenyl-lactoside layer.

The thickness of phenylglycoside layers was characterized via atomic force microscopy (AFM) using previously reported methods^31^,^32^. Briefly, phenylglycoside-modified surfaces were first imaged in tapping mode; subsequently, a section of the film was removed by scratching the sample with the AFM tip in contact mode; finally, the step created in the organic film was imaged in tapping mode. Figure 4 shows an example of a Lac-C surface imaged after the scratching process and of a height profile across the step. Height profiles were used to obtain an average thickness which was found to be 0.8 ± 0.1 nm in the case of both Gal-C and Lac-C layers. These thickness values are slightly lower than estimates of molecular length of 1.0 nm and 1.5 nm for phenyl-β-galactoside and benzyl-β-lactoside conformers, respectively, obtained from optimized glycoside geometries^33^,^34^. Thickness results therefore indicate that layers prepared via aryldiazonium chemistry using both mono- and di-saccharide groups reach a surface coverage of at most 1 monolayer, as expected based on the presence of bulky terminal groups such as Lac and Gal glycans^35^.

**Figure 5:**
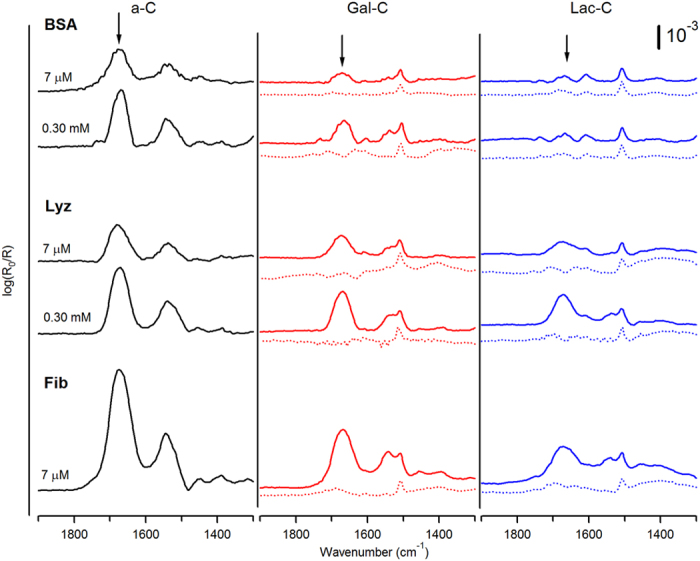
IRRAS spectra in the amide I/II region of bare a-C (black), Gal-C (red) and Lac-C (blue) surfaces after functionalization (dotted lines) and after incubation in buffered solutions of BSA, Lyz and Fib at different concentrations (solid lines). The position of the amide I band is indicated with an arrow.

In order to evaluate the antifouling properties of glycosylated interfaces, both unmodified and modified a-C substrates were incubated in buffered protein solutions for 1 h and analyzed by IRRAS *ex situ*. Three proteins with different properties were chosen for our studies: BSA, lysozyme (Lyz) and fibrinogen (Fib); a summary of their main physical properties is reported in Table 1 36,37,38. Figure 5 shows IRRAS spectra in the region 1900–1300 cm^−1^ of bare a-C, Gal-C and Lac-C surfaces after incubation in BSA, Lyz and Fib solutions at two different concentrations; dotted lines in the central and right hand panel show the IRRAS spectra of Gal-C and Lac-C surfaces prior to protein adsorption in the same spectral region. After adsorption, spectra exhibit the characteristic bands of amide groups in polypeptides: the two strong, broad peaks at ~1675 cm^−1^ and ~1540 cm^−1^ are assigned to the amide I and II modes, respectively^30^. Noticeably, the sharp peak at ~1510 cm^−1^ assigned to the aromatic ring appears in all of the spectra, thus confirming that the phenyl groups used for surface modification are strongly bound to the surface and are not displaced by adsorbed proteins. Similar results were obtained in the case of Man-C, Glc-C and Rha-C surfaces.

**Figure 6:**
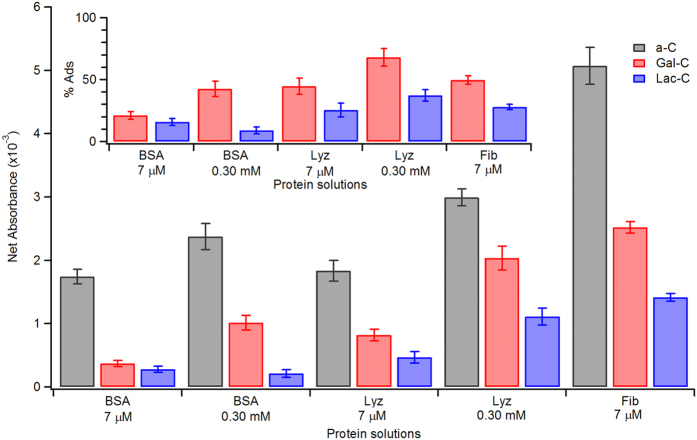
Comparison of amide I net absorbance values at a-C, Gal-C and Lac-C surfaces after incubation in solutions of BSA, Lyz and Fib. Inset shows adsorbed amounts relative to bare a-C surfaces.

The relative amounts of protein adsorbed at bare and saccharide-modified surfaces can be estimated from the net absorbance of amide bands in IRRAS spectra, under the assumption of no preferential orientation of peptide bonds at the carbon surface. Net absorbance values of amide I peaks at bare a-C, and phenylglycoside-modified carbon are reported in Fig. 6, where the inset shows the same results as percentage adsorption with respect to the bare surface. Values in Fig. 6 were obtained from adsorption experiments carried out at two different molar concentrations: 7 μM, equivalent to 0.5, 0.1 and 2.5 g L^−1^ for BSA, Lyz and Fib, respectively, and 0.30 mM, equivalent to 20 and 4.3 g L^−1^ for BSA and Lyz, respectively. These two concentrations are relevant for understanding the behavior of surfaces in physiological conditions since molar concentrations of 7 μM are in the normal range for Fib in plasma, while a 0.30 mM concentration is close to that of albumin in serum^39^. Fib could not be studied at the higher concentration because it falls beyond its solubility limit^40^.

IRRAS results indicate that at bare a-C surfaces, adsorption increases with increasing molar concentration for the same protein. Fib solutions yielded the strongest adsorption among all protein solutions tested. These observed trends are in general agreement with previous reports of adsorption isotherms of human albumin and fibrinogen at isotropic carbon surfaces by Feng and Andrade^41^. Adsorption values on monosaccharide-modified surfaces were significantly lower than at bare a-C for all three proteins at all concentrations studied. Similar results were obtained for surfaces modified with Glc, Man and Rha units: only small differences were observed in protein resistance among the four monosaccharides used in our studies. The amount of protein adsorbed at Lac-C was however found to be significantly lower than at either bare a-C, or monosaccharide-modified surfaces, thus indicating that Lac-C surfaces are better at resisting protein adsorption.

In order to obtain quantitative estimates of protein adsorption at mono- and disaccharide modified surfaces, Quartz Crystal Microbalance (QCM) measurements of protein mass were also carried out *ex situ*. Upon incubation in 7 μM BSA for 1 h, bare a-C surfaces reported a mass increase of 1.02 ± 0.27 μg cm^−2^, whereas Gal-C and Lac-C surfaces yielded increases of only (0.35 ± 0.22) and (0.10 ± 0.11) μg cm^−2^ (C.I. 95%), respectively. The above estimates likely constitute upper boundaries for BSA adsorption at the three surfaces, given that *ex situ* QCM also measures contributions from the mass of water trapped within the BSA layer. Table 2 summarizes BSA mass densities and relative adsorption mass values measured *via ex situ* QCM, together with the corresponding adsorption estimates obtained from amide I peak absorptions in IRRAS spectra. The comparison between the spectroscopic and gravimetric determination of protein adsorption was found to be satisfactory, thus indicating that amide I peak intensities are proportional to surface mass density of proteins on these surfaces. Also, gravimetric analysis confirms that Lac-C layers perform better than Gal-C layers in terms of protein rejection.

### Surface contact angle and surface free energy studies

Surface free energy (SFE) and wettability play an important role in defining the extent to which a surface can resist biofouling. The SFE of unmodified and modified a-C substrates was determined via contact angle (CA) measurements of multiple solvents using the sessile drop method. In order to obtain the SFE, we used the model of van Oss, Chaudhury and Good (vOCG)^42^,^43^. This model assumes that the total surface tension results from additive contributions of apolar, or Lifshitz-van der Waals (*γ*^*LW*^), and polar forces (*γ*^*AB*^):


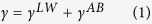


where *γ*^*AB*^ includes contributions *γ*^−^ and *γ*^+^ from electron donor-acceptor interactions, respectively, also called Lewis base-acid interactions. The model assumes that the work of adhesion at the solid-liquid interface, *W*_*SL*_ , can be approximated by geometric means as below:


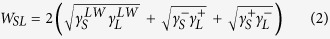


where the subscripts “*L*” and “*S*” indicate components of the liquid and solid, respectively. vOCG is considered to be a suitable model for describing the asymmetric nature of polar interactions when hydrogen bonding contributions are present^42^,^43^: electron donating and accepting groups must interact “reciprocally” to contribute to surface tension, as reflected by mixed donating/accepting products in Equation (2). Equation (2), in combination with the Young-Dupre equation results in:


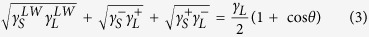


which can be used to obtain 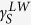, 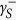 and 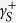 by measuring the CA of three liquids with known surface tension components 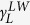, 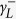 and 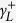.

Carbon films used for CA measurements were deposited on Si wafers and were found to display low rms roughness before and after modification (see Supporting Information). Surface tension components of the three test liquids at 20 °C are taken from van Oss’s data compilation^43^ and are reported in Table 3; the choice of liquids was based on the analysis of solvent triplets by Della Volpe *et al*^44^. 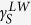 was first calculated using eq. (3) and the CA of diiodomethane, a liquid with 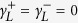. CAs of water and glycerol were then used to set a system of two linear equations that were solved for 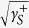 and 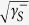
45; positive values were obtained from our calculations thus confirming that all surfaces yield physical solutions for 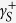 and 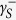.

CA values and surface tension components for all surfaces examined in this work are reported in Table 4. Bare a-C displayed a water CA of 35.3°, total SFE *γ*_*S*_ = 63.7 mJ m^−2^ and components 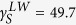 mJ m^−2^ and 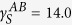 mJ m^−2^. These values are in good agreement with those reported by Leezenberg *et al*.^46^ for sputtered a-C films, but the polar component and total surface energy are higher than those obtained by Zebda *et al*.^45^ via vOCG analysis. Differences in components and total SFE could arise due to variations in material properties (e.g. sp^2^/sp^3^ or O-content) or film history^46^. Surface modification with saccharides leads to a significant decrease in water CA for all saccharide units tested, with the lowest CA observed for Lac-C surfaces. The total SFEs of phenylglycoside layers are slightly higher than that of bare a-C (<3% difference), with negligible differences observed among saccharides. Similarly, the apolar *γ*^*LW*^ contribution does not change significantly with surface treatment, remaining approximately constant across all surfaces (<3% difference). The most striking differences among surface modifications were observed in the acid-base components. The vOCG model does not permit to directly compare the solid acid/base contributions of a solid surface^44^; however, as discussed by Della Volpe *et al*.^44^, using the same solvent triplet it is possible to examine relative changes in acid and basic character brought upon by the surface modifications studied. Bare a-C displays the minimum 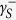 value; modification with monosaccharides increases surface basicity by 30–40%, and a further and significant increase is observed when the disaccharide Lac is used. This result is surprising as carbohydrate units are typically classified as hydrogen bond donors and thus would not be expected to increase the Lewis basicity of a surface; possible explanations for these findings are included in the Discussion section.

### Surface charge density at bare and modified carbon surfaces

Electrostatic interactions can play an important role in protein adsorption phenomena given that proteins and most surfaces possess ionizable groups whose charge is dependent on pH. To investigate whether electrostatic interactions could contribute to observed changes in protein adsorption upon carbon modification, we carried out ζ-potential measurements using standard solutions of tracer particles. Table 4 summarizes ζ-potential results obtained for a-C, Gal-C and Lac-C surfaces in 1 mM NaCl solutions at pH 9.2. The ζ-potential of a-C was found to be −55 ± 3 mV, whereas surface modification with phenylglycosides led to ζ-potential values for Gal-C and Lac-C of −56.3 ± 1.9 mV and −58.0 ± 2.6 mV, respectively.

These results indicate that surface modification via aryldiazonium chemistry results in negligible changes in ζ-potential with respect to that of the bare a-C substrate. This indicates that that functionalisation with phenylglycosides via this methodology offers a route for increasing the wettability of carbon surfaces without the introduction of significant changes in electrostatic charge, as is often the case with other modifications (e.g. oxidation). The implications of these results for understanding the origin of protein antifouling properties of aryldiazonium carbohydrate layers and for the design of carbohydrate coatings with enhanced antifouling properties will be discussed in the following section.

## Discussion

Protein adsorption studies on phenylglycoside layers obtained via aryldiazonium chemistry show that this functionalisation strategy leads to the formation of glycoside adlayers that impart resistance to protein adsorption. Spectroscopic and gravimetric studies carried out *ex situ*, all indicate that coated surfaces adsorb less protein than the unmodified carbon, with phenyl-lactoside groups appearing to be particularly effective at reducing unspecific adsorption.

Solvation/hydration forces have been identified as important for determining protein adsorption trends, given that solvation and desolvation processes play a key role in protein adsorption^47^. Many studies^1^,^7^,^21^,^47^,^48^,^49^,^50^ have in fact concluded that highly hydrophilic surfaces tend to prevent unspecific protein adsorption, whereas hydrophobic surfaces are more likely to favor protein adsorption because they are easier to dehydrate and because they can maximize their interactions with protein hydrophobic groups through changes in protein secondary structure upon adsorption^51^. In the case of aryldiazonium carbohydrate layers, CA measurements indicate that modification results in greater hydrophilicity; this correlates well with the reduction in protein adsorption that was observed in general, for all the three proteins at both concentration ranges examined. Lac-C surfaces were found to be the most effective carbohydrate-modified surfaces in terms of repelling protein fouling, and the ones with the lowest water CA in agreement with trends that positively correlate wettability with protein resistance.

The contributions of polar and dispersive interactions resulting in the observed wettability were obtained from a multisolvent determination and analysis of Surface Free Energies (SFE). Carbohydrate surfaces obtained via aryldiazonium chemistry possess SFEs that are <3% higher than that of a-C. However the analysis based on the vOCG model suggests that large differences are introduced in the polar contributions to the total SFE, via modification of carbon with phenylglycosides. The solid-water interfacial SFE can be estimated from the data in Table 4, according to 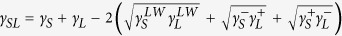, which yields values of 4.3, −0.2 and −6.6 mJ m^−2^ for a-C, Gal-C and Lac-C surfaces, respectively. The observation of decreasing fouling in the order a-C > Gal-C > Lac-C is therefore consistent with expectations based on values of *γ*_*SL*_ calculated from CA results.

Analysis of SFE components also indicates that surface modification via aryldiazonium phenyl-glycosides increases the Lewis basicity of the carbon surface: Glc-C, Man-C, Gal-C and Rha-C have 30–40% greater 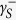 values than that of bare a-C, while phenyl-lactoside immobilization leads to a 60% increase. This is somewhat surprising as carbohydrate units are typically classified as hydrogen bond donors and, thus, would not be expected to increase the Lewis basicity of a surface. Evidence from studies on alkylthiols indicates that the presence of groups that are polar, neutral and hydrogen-bond acceptors promotes fouling resistance^21^,^52^. Carbohydrates have been identified as exceptions to the hydrogen-acceptor requirement, however vOCG results suggest that this might not be the case and that once carbohydrates are immobilized they can actually enhance the hydrogen-acceptor character of surfaces. We speculate that saccharide-saccharide and saccharide-water intermolecular bonding within a dense glycan layer, might result in the basicity displayed by phenylglycoside layers. It is likely that engagement of hydroxyl groups in intra-layer hydrogen bonding modulates the hydrogen bonding properties displayed by the saccharide layer at the interface.

Çarçabal *et al*.^33^ carried out experimental and computational work on Man, Gal and Glc phenylglycosides and on benzyl-β-lactoside in the gas phase, showing that hydration leads to the formation of extended intra- and intermolecular hydrogen bond networks. The effect of hydration was greater in the case of benzyl-β-lactoside which was found to effectively lock into conformation through cooperative hydrogen bonding. It appears therefore likely that the water shroud associated with saccharide units would create a barrier to dehydration, and contributes to the protein resistance of carbohydrate aryldiazonium coatings. Further studies that directly probe hydrogen bonding within aryldiazonium layers would be desirable, to determine whether trends observed for phenylglycosides in the gas phase also translate to thin films of surface-immobilized groups.

Finally, the surface-blocking effect and the steric hindrance of the saccharide moiety in phenylglycoside layers is likely to also contribute to preventing adsorption of proteins, given that coatings displaying bulky groups can screen protein-substrate interactions. Molecular density however might play a role beyond blocking access to the carbon surface, by also regulating the observed basicity of saccharide layers through intermolecular interactions within the adlayer. Thus it would be important in future studies to identify whether the observed basicity and protein resistance behavior vary significantly with molecular surface density, given the same carbohydrate motif. Conversely, carbohydrate structure might be leveraged to enhance or reduce hydrogen bonding by selecting units with different propensity to engage in inter/intra molecular hydrogen bonding. Studies of layers prepared with oligosaccharide moieties that display predominantly inter- or intra-chain bonding might reveal more about the role of inter and intra-chain interactions in determining basicity and protein fouling resistance in phenylglycoside layers.

## Conclusions

We have investigated the adsorption of three proteins at carbon surfaces modified with phenylglycoside layers prepared via aryldiazonium chemistry; layers bearing both monosaccharides and a di-saccharide, lactose, were prepared and compared in their properties and protein resistance to bare carbon surfaces. Results indicate that these coatings display good protein resistance and that judicious choice of synthetic phenylglycosides can be used to optimize resistance. This is an important finding from a practical standpoint because aryldiazonium covalent immobilization is a versatile method for the functionalization of carbons and nanocarbons. Furthermore, it is known to work with a wide range of substrate materials beyond carbon and it is applicable under mild conditions from dip, spray and contact deposition methods. Thus, the methodology offers a versatile route to imparting antifouling properties onto surfaces of complex, mixed material devices, e.g. for biosensing, implantation, blood contacting applications.

A study of interfacial physical properties revealed that the protein resistance of these layers correlates well with their hydrophilic character when compared to the bare carbon material. An increase in wettability with respect to bare carbon is achieved without a significant change in surface charge density. Interestingly, we notice that mono and di-saccharides increase the Lewis basicity of the surface, contrary to expectations from typical reactivity patterns of carbohydrates in solution. This finding is consistent with empirical rules on the type of properties that lead to protein fouling resistance of thin-organic layers. We propose that the observed basicity might arise from inter- and intra- molecular hydrogen bonding networks, which could alter the acid-base properties of units exposed at the surface. Further studies would be desirable for understanding the correlation between Lewis basicity and inter- and intra-molecular hydrogen bonding in the phenylglycoside layer. The vast number of existing carbohydrate structural motifs offers an exciting landscape for exploring the potential of these layers to leverage structural variability and achieve tunable fouling resistance.

## Experimental Methods

### Chemicals and Materials

Diiodomethane (99%), glycerol (≥99.5%), sulfuric acid (95–97%), hydrochloric acid (37%), hydrogen peroxide (30%), fluoroboric acid (48 wt.% in H_2_O), sodium nitrite (≥99.0%), acetonitrile (HPLC grade) and methanol (semiconductor grade) were purchased from Sigma and used without further purification. B-doped Si wafers were purchased from MicroChemicals and 10 MHz quartz crystals were purchased from International Crystal Manufacturing. Bovine Serum Albumin (BSA, ≥96%), Lysozyme from chicken egg white (Lyz), Fibrinogen from bovine plasma (Fib, 65–85% protein) and phosphate saline buffer tablets (PBS, 0.01 M, 0.0027 KCl and 0.137 NaCl pH 7.4) were purchased from Sigma. Millipore water was used for all experiments. Precursors 4-aminophenol-β-D-glucoopyranose (**1**), 4-aminophenol-β-D-galactopyranose (**2**), 4-aminophenol-α-D-mannopyranose (**3**), 4-aminophenol-α-L-rhamnopyranose (**4**) and 4-aminophenol-β-D-lactopyranose (**5**) (see Fig. 2) were synthesized as previously reported^20^,^27^.

### Substrate preparation

Amorphous carbon films (a-C) with thickness 73.6 ± 0.6 nm (C.I. 95%) were prepared via DC magnetron sputtering (Torr International, Inc.) at a base pressure ≤2 × 10^−6^ mbar and a deposition Ar pressure of 7 × 10^−3^ mbar, as previously described^29^. Silicon wafers were cleaned in piranha solution prior to deposition (H_2_SO_4_ : H_2_O_2_ in a 3:1 ratio – *WARNING: Piranha solution is a strong oxidant and reacts violently with organic materials and presents an explosion danger; all work should be performed under a fume hood*). For infrared reflectance absorbance spectroscopy (IRRAS) measurements, Si wafers were coated prior to a-C deposition, with an optically thick (449 ± 29) nm (C.I. 95%) Ti layer via DC magnetron sputtering. Surface modification with carbohydrate moieties was carried out as previously reported^27^, and following a protocol summarized in Fig. 1. Briefly, 4-aminophenyl glycosides were dissolved in acid; while keeping the solution in an ice bath, NaNO_2_ was added yielding the corresponding aryldiazonium salt *in situ* at a final concentration of 1.0 mM. Carbon samples were immersed in the aryldiazonium salt solution for 1 h, rinsed in acetonitrile and methanol and dried under argon prior to further use.

### Characterization Methods

Static contact angles (CA) were measured on a CA analyzer (FTA) under ambient conditions of temperature and humidity; samples were rinsed in methanol immediately prior to CA characterization^45^ and a minimum of three CA measurements were obtained for each surface. Spectroscopic Ellipsometry (SE) was carried out using an alpha-SETM ellipsometer (J.A. Woolam Co.). a-C films were deposited on clean Si wafers and measured at 65°, 70°, 75° incidence angle over the 370–900 nm range; SE data was then fitted using the CompleteEASE® software package using a three layer model to account for Si, a-C and air phases (see Supporting Information). ζ-potential measurements were carried out using a Malvern Zetasizer Nano-ZS equipped with a surface ζ-potential cell; standard 300 nm latex tracer particle suspensions, NaCl 1 mM, at pH 9.2 (Malvern, DTS1235) were used in all experiments. IRRAS was carried out on a Fourier Transform Infrared (FTIR) spectrometer (Tensor 27, Bruker) equipped with a Mercury Cadmium Telluride (MCT) detector, a specular reflectance accessory (VeeMax II), and a ZnSe polarizer. Spectra were taken at 80° incidence using p-polarized light; 100 spectra were collected at 4 cm^−1^ resolution using a bare substrate as background. All spectra reported in this work were baseline corrected using commercial FTIR software (WinFIRST). Quartz Crystal Microbalance (QCM) measurements were carried out *ex situ* following a previously reported procedure^27^. The resonant frequency of a carbon coated QCM crystal was measured in air before and after protein adsorption, and the difference was used to calculate the mass change at the crystal via the Sauerbrey equation^53^. Measurements were carried out in a home-built chamber at the same temperature before and after modification; in the case of lactose-modified surfaces it was necessary to introduce a dessicant (Drierite®) in the measurement chamber in order to achieve frequency stability, likely due to water adsorption by surface-bound disaccharide units. Thickness and surface roughness measurements were carried out via Atomic Force Microscopy (AFM, Asylum Research) using silicon catilevers.

### Protein adsorption experiments

BSA, Lyz and Fib were dissolved in 0.01 M PBS buffer (pH 7.4) at different concentrations for each protein: 0.5 and 20 mg/mL for BSA, 0.1 and 4.3 mg/mL for Lyz and 2.5 mg/mL for Fib. Carbohydrate-coated and bare a-C surfaces were incubated in buffered protein solutions for 1 h at ambient temperature (20 °C). Substrates were rinsed, immersed for 10 min in water, and finally dried under argon prior to characterization.

## Additional Information

**How to cite this article**: Zen, F. *et al*. Modulation of Protein Fouling and Interfacial Properties at Carbon Surfaces via Immobilization of Glycans Using Aryldiazonium Chemistry. *Sci. Rep.* **6**, 24840; doi: 10.1038/srep24840 (2016).

## Supplementary Material

Supplementary Information

## Figures and Tables

**Table 1:** Main properties of proteins used for adsorption studies; molar mass, number of amino acids and isoelectric point are provided by the manufacturer, except for the isoelectric point of Fib which is taken from ref. 36 and sizes which are taken from refs 37 and 38.

	Mass (kDa)	Amino acids	Size (nm^3^)	Isoelectric point
BSA	66	583	8 × 8.7 × 6	4.7–4.9
Lyz	14	129	4.5 × 3 × 3	11.35
Fib	340	3620	45 × 9 × 6	5.8

**Table 2:** BSA adsorption measurements at a-C, Gal-C and Lac-C surfaces, carried out using 7 μM solutions.

Surface	Adsorbed BSA (μg cm^−2^)	Relative BSA Mass	Relative Amide I peak absorbance
a-C	1.02 ± 0.27	–	–
Gal-C	0.35 ± 0.22	34%	21%
Lac-C	0.10 ± 0.11	9.8%	16%

The table reports absolute adsorbed mass values determined via *ex situ* QCM, relative adsorbed masses calculated with respect to adsorption at bare a-C and relative adsorbed values determined via IRRAS under the same experimental conditions.

**Table 3:** Total surface tensions (***γ***_***L***_), dispersive ( 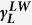 ), electron donating 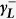 and accepting ( 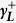 ) components (mJ m^−2^) of test liquids used for contact angle measurements and vOCG analysis^43^,^45^.

Test liquids	*γ*_*L*_	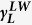	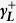	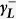
Water	72.8	21.8	25.5	25.5
Glycerol	63.3	34	3.92	57.4
Diiodomethane	50.8	50.8	0	0

**Table 4:** Summary of experimentally determined properties of bare and modified carbon surfaces: measured contact angles using water (**θ**_**W**_), glycerol (**θ**_**G**_) and diiodomethane (**θ**_**DM**_); surface free energy or total surface tension (***γ***_***tot***_), its dispersive (***γ*** ^***LW***^), electron accepting (***γ*** ^**+**^) and donating (***γ*** ^**−**^) components determined from vOCG analysis; surface ζ-potential values obtained using polystyrene tracer particles in 1 mM NaCl at pH 9.2.

Surface	Contact Angles (degrees)			SFE components (mJ m^−2^)				
	θ_W_	θ_G_	θ_DM_	*γ*^*LW*^	*γ*^+^	*γ*^−^	*γ*_*tot*_	ζ-potential (mV)
a-C	35.3 ± 1.4	22.2 ± 1.4	11.9 ± 0.9	49.7	1.79	27.4	63.7	−55 ± 3
Glc-C	20.2 ± 0.3	16.4 ± 1.2	5.4 ± 0.4	50.6	1.38	38.7	65.2	–
Gal-C	26.1 ± 1.1	17.1 ± 0.4	7.5 ± 0.4	50.4	1.61	34.2	65.2	−56.3 ± 1.9
Man-C	22.9 ± 0.6	15.9 ± 1.3	4.9 ± 0.1	50.6	1.52	36.5	65.5	–
Rha-C	25.0 ± 0.5	20.3 ± 0.6	6.2 ± 0.2	50.4	1.30	36.3	64.2	–
Lac-C	11.8 ± 0.4	16.9 ± 0.7	4.2 ± 0.3	50.7	1.11	43.9	64.6	−58.0 ± 2.6

## References

[b1] Ratner B. D., Hoffman A. S., Schoen F. J. & Lemons J. E. Biomaterials Science. 2^nd^ edn, (Elsevier Academic Press, 2004).

[b2] Andrade J. D., Hlady V., Feng L. & Tingey K. In Interfacial phenomena and bioproducts Bioprocess Technology (eds Brash J. L. & Wojciechowski P. W.) Ch. 2, 19–55 (Marcel Dekker, 1996).

[b3] Monopoli M. P., Aberg C., Salvati A. & Dawson K. A. Biomolecular coronas provide the biological identity of nanosized materials. Nat Nano 7, 779–786 (2012). 10.1038/nnano.2012.207 23212421

[b4] Aggarwal P., Hall J. B., McLeland C. B., Dobrovolskaia M. A. & McNeil S. E. Nanoparticle interaction with plasma proteins as it relates to particle biodistribution, biocompatibility and therapeutic efficacy. Adv. Drug Delivery Rev. 61, 428–437 (2009). 10.1016/j.addr.2009.03.009 PMC3683962 19376175

[b5] Cifuentes-Rius A., de Puig H., Kah J. C. Y., Borros S. & Hamad-Schifferli K. Optimizing the Properties of the Protein Corona Surrounding Nanoparticles for Tuning Payload Release. ACS Nano 7, 10066–10074 (2013). 24128271 10.1021/nn404166q

[b6] Roy D. Surface Plasmon Resonance Spectroscopy of Dielectric Coated Gold and Silver Films on Supporting Metal Layers: Reflectivity Formulas in the Kretschmann Formalism. Appl. Spectrosc. 55, 1046–1052 (2001).

[b7] Stueber M. . Surface topography, surface energy and wettability of magnetron-sputtered amorphous carbon (a-c) films and their relevance for platelet adhesion. Adv. Eng. Mater. 9, 1114–1122 (2007).

[b8] Lu F. . Advances in Bioapplications of Carbon Nanotubes. Adv. Mater. 21, 139–152 (2009).

[b9] Mochalin V. N., Shenderova O., Ho D. & Gogotsi Y. The properties and applications of nanodiamonds. Nat Nano 7, 11–23 (2012). 10.1038/nnano.2011.209 22179567

[b10] McCreery R. L. Advanced Carbon Electrode Materials for Molecular Electrochemistry. Chem. Rev. 108, 2646–2687 (2008). 18557655 10.1021/cr068076m

[b11] Lockett M. R. & Smith L. M. Carbon Substrates: A Stable Foundation for Biological Arrays. Annu. Rev. Anal. Chem. 8, 17.11–17.23 (2015). 10.1146/annurev-anchem-071114-040146 PMC6287745 26048550

[b12] Clare T. L., Clare B. H., Nichols B. M., Abbott N. L. & Hamers R. J. Functional Monolayers for Improved Resistance to Protein Adsorption: Oligo (ethylene glycol)-Modified Silicon and Diamond Surfaces. Langmuir 21, 6344–6355 (2005). 15982041 10.1021/la050362q

[b13] Trouillon R., O’Hare D. & Einaga Y. Effect of the doping level on the biological stability of hydrogenated boron doped diamond electrodes. Phys. Chem. Chem. Phys. 13, 5422–5429 (2011). 21380425 10.1039/c0cp02420a

[b14] Mu Q. . Chemical Basis of Interactions Between Engineered Nanoparticles and Biological Systems. Chem. Rev. 114, 7740–7781 (2014). 24927254 10.1021/cr400295a PMC4578874

[b15] Harrison C. Nanotechnology: Biological proteins knock nanoparticles off target. Nat Rev Drug Discov 12, 264–264 (2013). 23493078 10.1038/nrd3983

[b16] Banerjee I., Pangule R. C. & Kane R. S. Antifouling Coatings: Recent Developments in the Design of Surfaces That Prevent Fouling by Proteins, Bacteria, and Marine Organisms. Adv. Mater. 23, 690–718 (2011). 20886559 10.1002/adma.201001215

[b17] Ostuni E. . Self-assembled monolayers that resist the adsorption of proteins and the adhesion of bacterial and mammalian cells. Langmuir 17, 6336–6343 (2001).

[b18] Hilkens J., Ligtenberg M. J. L., Vos H. L. & Litvinov S. V. Cell membrane-associated mucins and their adhesion-modulating property. Trends Biochem. Sci. 17, 359–363 (1992). 1412714 10.1016/0968-0004(92)90315-z

[b19] Sumiyoshi M. . Antiadhesive Character of Mucin O-glycans at the Apical Surface of Corneal Epithelial Cells. Invest. Ophthalmol. Vis. Sci. 49, 197–203 (2008). 18172093 10.1167/iovs.07-1038 PMC2247472

[b20] Angione M. D. . Enhanced antifouling properties of carbohydrate coated poly (ether sulfone) membranes. ACS Appl. Mater. Interfaces 7, 17238–17246 (2015). 26192984 10.1021/acsami.5b04201

[b21] Ederth T. . Resistance of Galactoside-Terminated Alkanethiol Self-Assembled Monolayers to Marine Fouling Organisms. ACS Appl. Mater. Interfaces 3, 3890–3901 (2011). 21916438 10.1021/am200726a

[b22] Österberg E. . Comparison of polysaccharide and poly (ethylene glycol) coatings for reduction of protein adsorption on polystyrene surfaces. Colloids Surf A Physicochem Eng Asp. 77, 159–169 (1993).

[b23] Holland N. B., Qiu Y. X., Ruegsegger M. & Marchant R. E. Biomimetic engineering of non-adhesive glycocalyx-like surfaces using oligosaccharide surfactant polymers. Nature 392, 799–801 (1998). 9572137 10.1038/33894

[b24] Perrino C., Lee S., Choi S. W., Maruyama A. & Spencer N. D. A Biomimetic Alternative to Poly (ethylene glycol) as an Antifouling Coating: Resistance to Nonspecific Protein Adsorption of Poly (l-lysine)-graft-dextran. Langmuir 24, 8850–8856 (2008). 18616303 10.1021/la800947z

[b25] Lord M. S., Pasqui D., Barbucci R. & Milthorpe B. K. Protein Adsorption on Derivatives of Hyaluronan. Macromol. Symp. 266, 17–22 (2008).

[b26] Mussard W., Kebir N., Kriegel I., Estève M. & Semetey V. Facile and Efficient Control of Bioadhesion on Poly (dimethylsiloxane) by Using a Biomimetic Approach. Angew. Chem. Intl. Ed. 50, 10871–10874 (2011). 10.1002/anie.201101029 21948488

[b27] Jayasundara D. R. . Carbohydrate Coatings via Aryldiazonium Chemistry for Surface Biomimicry. Chem. Mater. 25, 4122–4128 (2013).

[b28] Barriere F. & Downard A. J. Covalent modification of graphitic carbon substrates by non-electrochemical methods. J. Solid State Electrochem. 12, 1231–1244 (2008).

[b29] Cullen R. J. . Spontaneous grafting of nitrophenyl groups on amorphous carbon thin films: A structure-reactivity investigation. Chem. Mater. 24, 1031–1040 (2012).

[b30] Socrates G. Infrared and Raman Characteristic Group Frequencies: Tables and Charts. (John Wiley & Sons, 2001).

[b31] Brooksby P. A. & Downard A. J. Electrochemical and Atomic Force Microscopy Study of Carbon Surface Modification via Diazonium Reduction in Aqueous and Acetonitrile Solutions. Langmuir 20, 5038–5045 (2004). 15984266 10.1021/la049616i

[b32] Anariba F., DuVall S. H. & McCreery R. L. Mono- and Multilayer Formation by Diazonium Reduction on Carbon Surfaces Monitored with Atomic Force Microscopy “Scratching”. Anal. Chem. 75, 3837–3844 (2003). 14572051 10.1021/ac034026v

[b33] Çarçabal P. . Hydrogen Bonding and Cooperativity in Isolated and Hydrated Sugars: Mannose, Galactose, Glucose, and Lactose. J. Am. Chem. Soc. 127, 11414–11425 (2005). 16089470 10.1021/ja0518575

[b34] Jockusch R. A. . Probing the Glycosidic Linkage: UV and IR Ion-Dip Spectroscopy of a Lactoside. J. Am. Chem. Soc. 126, 5709–5714 (2004). 15125663 10.1021/ja031679k

[b35] Combellas C., Jiang D.-e., Kanoufi F., Pinson J. & Podvorica F. I. Steric Effects in the Reaction of Aryl Radicals on Surfaces. Langmuir 25, 286–293 (2009). 19067511 10.1021/la8025792

[b36] Wasilewska M., Adamczyk Z. & Jachimska B. Structure of Fibrinogen in Electrolyte Solutions Derived from Dynamic Light Scattering (DLS) and Viscosity Measurements. Langmuir 25, 3698–3704 (2009). 19228031 10.1021/la803662a

[b37] Höök F. . A comparative study of protein adsorption on titanium oxide surfaces using *in situ* ellipsometry, optical waveguide lightmode spectroscopy, and quartz crystal microbalance/dissipation. Colloids Surf., B 24, 155–170 (2002).

[b38] Pasche S., Vörös J., Griesser H. J., Spencer N. D. & Textor M. Effects of Ionic Strength and Surface Charge on Protein Adsorption at PEGylated Surfaces. J. Phys. Chem. B 109, 17545–17552 (2005). 16853244 10.1021/jp050431+

[b39] Kratz A., Ferraro M., Sluss P. M. & Lewandrowski K. B. Normal Reference Laboratory Values. N. Engl. J. Med. 351, 1548–1563 (2004). 15470219 10.1056/NEJMcpc049016

[b40] Rampling M. W. The solubility of fibrinogen in solutions containing dextrans of various molecular weights. Biochem. J. 143, 767–769 (1974). 4462754 10.1042/bj1430767 PMC1168446

[b41] Feng L. & Andrade J. D. Protein adsorption on low temperature isotropic carbon: III. Isotherms, competitivity, desorption and exchange of human albumin and fibrinogen. Biomaterials 15, 323–333 (1994). 8061122 10.1016/0142-9612(94)90243-7

[b42] Van Oss C. J., Chaudhury M. K. & Good R. J. Interfacial Lifshitz-van der Waals and polar interactions in macroscopic systems. Chem. Rev. 88, 927–941 (1988).

[b43] van Oss J. C. Interfacial Forces in Aqueous Media. 1st edn, (Marcel Dekker, 1994).

[b44] Della Volpe C., Maniglio D., Brugnara M., Siboni S. & Morra M. The solid surface free energy calculation: I. In defense of the multicomponent approach. J. Colloid Interface Sci. 271, 434–453 (2004). 14972623 10.1016/j.jcis.2003.09.049

[b45] Zebda A., Sabbah H., Ababou-Girard S., Solal F. & Godet C. Surface energy and hybridization studies of amorphous carbon surfaces. Appl. Surf. Sci. 254, 4980–4991 (2008).

[b46] Leezenberg P. B., Johnston W. H. & Tyndall G. W. Chemical modification of sputtered amorphous-carbon surfaces. J. Appl. Phys. 89, 3498–3507 (2001).

[b47] Kasemo B. Biological surface science. Surf. Sci. 500, 656–677 (2002).

[b48] Kirschner C. M. & Brennan A. B. Bio-Inspired Antifouling Strategies. Annu. Rev. Mater. Res. 42, 211–229 (2012).

[b49] Morra M. Water in Biomaterials Surface Science (John Wiley & Sons, Baffins Lane, Chichester, 2001).

[b50] Ham H. O., Park S. H., Kurutz J. W., Szleifer I. G. & Messersmith P. B. Antifouling Glycocalyx-Mimetic Peptoids. J. Am. Chem. Soc. 135, 13015–13022 (2013). 23919653 10.1021/ja404681x PMC3807125

[b51] Feng L. & Andrade J. D. Protein adsorption on low temperature isotropic carbon. 1. Protein conformational change probed by differential scanning calorimetry. J. Biomed. Mater. Res. 28, 735–743 (1994). 8071385 10.1002/jbm.820280611

[b52] Ostuni E., Chapman R. G., Holmlin R. E., Takayama S. & Whitesides G. M. A Survey of Structure−Property Relationships of Surfaces that Resist the Adsorption of Protein. Langmuir 17, 5605–5620 (2001).

[b53] Sauerbrey G. Z. Use of quartz vibration for weighing thin films on a microbalance. J. Physik 155, 206–212 (1959).

