# Supplementary material for: Modulation of Protein Fouling and Interfacial Properties at Carbon Surfaces via Immobilization of Glycans Using Aryldiazonium Chemistry
Source: Sci Rep. 2016 Apr 25;6:24840. doi: 10.1038/srep24840 (PMC4843010; doi:10.1038/srep24840)
Supplement: Supplementary Information [file srep24840-s1.pdf]

Supporting Information

Modulation of Protein Fouling and Interfacial Properties at Carbon Surfaces via Immobilization of Glycans Using Aryldiazonium Chemistry

Federico Zen, M. Daniela Angione, James A. Behan, Ronan J. Cullen, Thomas Duff, Joana M. Vasconcelos, Eoin M. Scanlan,* Paula E. Colavita*

School of Chemistry and Centre for Centre for Research on Adaptive Nanostructures and Nanodevices (CRANN), Trinity College Dublin, College Green, Dublin 2, Ireland

E-mail: eoin.scanlan@tcd.ie; colavitp@tcd.ie

1. Spectroscopic ellipsometry determination of a-C film thickness

Spectroscopic ellipsometry (SE) measurements of amorphous carbon films (a-C) deposited on undoped silicon wafers were taken using a J.A. Woolam Co. Inc. alpha-SETM ellipsometer at incident angles of 65^0, 70^0 and 75^0; all measurements were taken with standard sample mounting. Ψ and Δ experimental values were fitted using CompleteEASE® software (J.A. Woolam Inc.) following a procedure based on an approach previously reported in the literature by Weber et al.[1] The samples were modelled as four distinct layers: a Si semi-infinite substrate, a native SiO_2 layer, an a-C layer, and a 'void' layer representing the atmosphere above the sample. The Si substrate was first characterized separately via SE using optical constants for the Si and SiO_2 layers taken from Herzinger et al.;[2] the thickness of the native oxide was thus determined to be 1.9 nm and kept fixed at that value in all models used for subsequent thickness determinations. The a-C layer in the model stack was the only one allowed to vary and its optical constants were fitted using B-Splines while enforcing Kramers-Kronig consistency.[1] This represents a purely mathematical

parameterization i.e. it requires no knowledge of the film's properties but still results in optical constants that are physically meaningful. The B-spline fit yielded film thickness, as well as real and imaginary refractive indeces *n* and *k*. Figure S1 shows an example of the real and imaginary part of the refractive index determined via this method. Fits of multiple a-C films were carried out using this procedure yielding an average thickness of 73.6 ± 0.6 nm (C.I. 95%). A Tauc plot carried out using *k* values obtained from SE determinations yielded Tauc bandgaps of 0.85 ± 0.06 eV (C.I. 95%). The bandgap value is slightly higher than our previous determination via approximate transmittance measurements,[3] as expected based on the use of SE-determined *k* values, which are not affected by reflectance losses.

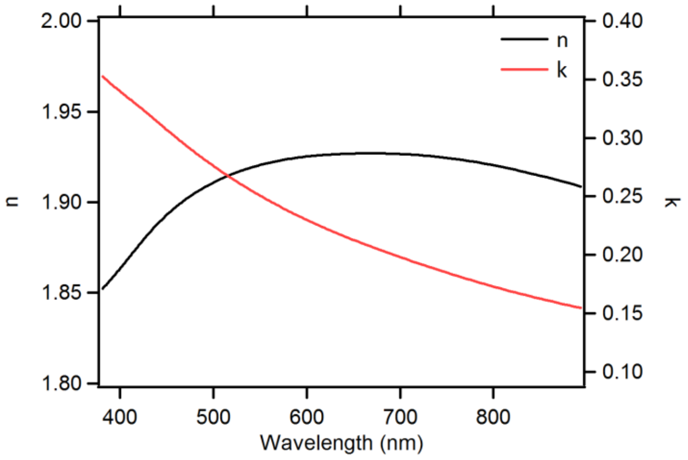

Figure S1: A graph of optical constants, *n* and *k*, of amorphous carbon films obtained via spectroscopic ellipsometry.

2. Atomic Force Microscopy determination of Ti substrate thickness

The thickness of Ti underlayers used for IRRAS spectroscopy was determined via Atomic Force Microscopy (AFM) in contact mode. Si wafers were sputtered with Ti under the conditions reported in the Experimental section in the main text; prior to sputtering the wafer was coated for half of its surface with a 2% solution of PMMA in anisole. After sputtering the PMMA was dissolved in acetone, thus creating a step edge on which AFM measurements were carried out yielding (449 ± 29) nm (C.I. 95%); the observed precision is consistent with typical uniformity of sputtered layers in the range 5-10%. Figure S2 shows an AFM image of the step used for Ti thickness determinations.

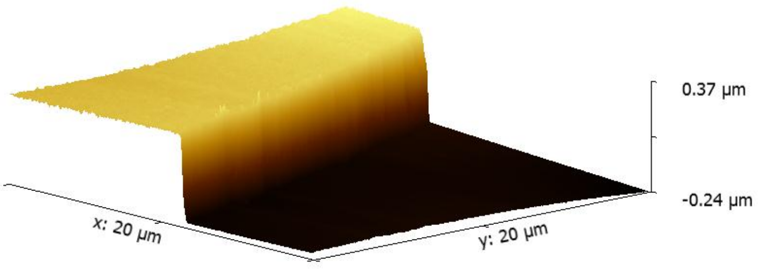

Figure S2: Step edge in a sputtered Ti layer used to measure the thickness of Ti underlayers.

3. RMS roughness determinations of bare and modified carbon surfaces

Roughness determinations were carried out via AFM in tapping mode, using Si cantilevers. 512 line images were collected and roughness was analysed using commercial software (Gwyddion) by defining a 10 × 10 μm^2 box in different regions of the sample surface. Figure S3 shows examples of topography images for bare and monosaccharide modified surfaces. Table S1 shows a summary of rms roughness values obtained via AFM which were found to be better than 1.5 nm over 100 μm^2.

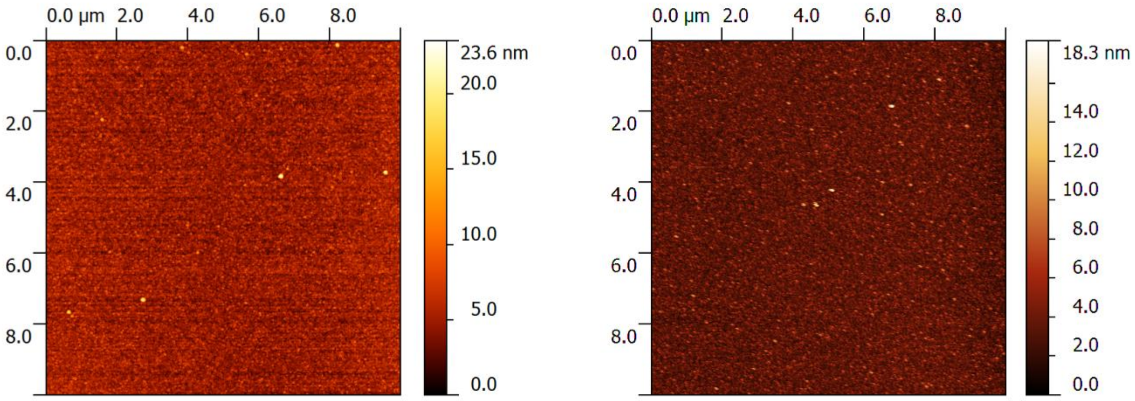

Figure S3: AFM topography images of bare a-C (left) and Lac-C (right) surfaces.

Sample	RMS roughness (nm)
Bare a-C	**1.45** ± 0.06
Gal-C	**1.17** ± 0.07
Rha-C	**1.10** ± 0.12
Man-C	**1.35** ± 0.05
Glc-C	**1.02** ± 0.04
Lac-C	**1.21** ± 0.03

Table S1: Average RMS roughness measured for bare and modified surfaces using tapping mode AFM.

References

1. Weber, J. W., Hansen, T. A. R., van de Sanden, M. C. M. & Engeln, R. B-spline parametrization of the dielectric function applied to spectroscopic ellipsometry on amorphous carbon. *J. Appl. Phys.* **106**, 123503, (2009).

2. Herzinger, C. M., Johs, B., McGahan, W. A., Woollam, J. A. & Paulson, W. Ellipsometric determination of optical constants for silicon and thermally grown silicon dioxide via a multi-sample, multi-wavelength, multi-angle investigation. *J. Appl. Phys.* **83**, 3323-3336, (1998).

3. Cullen, R. J. *et al.* Spontaneous grafting of nitrophenyl groups on amorphous carbon thin films: A structure-reactivity investigation. *Chem. Mater.* **24**, 1031-1040, (2012).
